# Prognosis of early pre-discharge and late left ventricular dilatation by cardiac magnetic resonance imaging after acute myocardial infarction

**DOI:** 10.1007/s10554-020-02136-5

**Published:** 2021-01-12

**Authors:** Martin R. Sinn, Gunnar K. Lund, Kai Muellerleile, Eric Freiwald, Maythem Saeed, Maxim Avanesov, Alexander Lenz, Jitka Starekova, Yskert von Kodolitsch, Stefan Blankenberg, Gerhard Adam, Enver Tahir

**Affiliations:** 1 Department of Diagnostic and Interventional Radiology and Nuclear Medicine, University Hospital Hamburg Eppendorf, Martinistr. 52, 20246 Hamburg, Germany; 2 Department of General and Interventional Cardiology, University Heart Center, Hamburg, Germany; 3 Department of Radiology and Biomedical Imaging, UCSF School of Medicine, 185 Berry Street, San Francisco, CA 94143 USA; 4 Institute for Medical Biometry and Epidemiology, University Hospital Hamburg Eppendorf, Hamburg, Germany

**Keywords:** Cardiac magnetic resonance imaging, Myocardial infarction, Left ventricle, Dilatation, Prognosis

## Abstract

To study the long-term prognosis of early pre-discharge and late left ventricular (LV) dilatation in patients with first ST-elevation myocardial infarction (STEMI) treated by percutaneous coronary intervention (PCI) and contemporary medical therapy. Long-term follow-up > 15 years was available in 53 consecutive patients (55 ± 13 years) with first STEMI. Late gadolinium enhanced (LGE) cardiac magnetic resonance imaging (CMR) was obtained at baseline 5 ± 3 days and follow-up 8 ± 3 months after STEMI to measure LV function, volumes and infarct size. Early pre-discharge dilatation was defined as increased left ventricular end-diastolic volume index (LVEDVi) at baseline CMR with > 97 ml/m^2^ for males and > 90 ml/m^2^ for females. Late dilatation was defined as initially normal LVEDVi, which increased ≥ 20% at follow-up. Early dilatation was present in 7 patients (13%), whereas late dilatation occurred in 11 patients (21%). Patients with early LV dilatation had highest mortality (57%), whereas patients with late dilatation had similar mortality (27%) compared to patients without dilatation (26%). Multivariate Cox analysis showed that age (*P* < 0.001), ejection fraction at baseline (*P* < 0.01) and early dilatation (*P* < 0.01) were independent predictors of death. Early dilatation qualified as an exclusive independent predictor of long-term mortality after adjustment for age and ejection fraction (*P* < 0.05, hazard ratio: 2.2, 95% confidence interval: 1.2 to 7.9). Early pre-discharge LV dilatation by CMR enabled strong long-term risk stratification after STEMI. The high mortality of early LV dilatation underscores the clinical importance of this post-infarction complication, which occurred despite PCI and contemporary medical therapy.

**Supplementary Information:**

The online version contains supplementary material available at 10.1007/s10554-020-02136-5.

## Introduction

Left ventricular (LV) dilatation after acute myocardial infarction (AMI) is a complex and dynamic process with at least two components. Early LV dilatation occurs within the first 72 h after AMI, it is characterized by LV enlargement and thinning of the infarct area which results from stretching of the infarct zone due to slippage of necrotic myofibrils [1–3]. Late LV dilatation occurs during the subsequent months after AMI. This dilatation type involves the entire ventricle leading to changes in LV shape and architecture in response to loss of myocardium and increased wall stress [4, 5]. Early and late LV dilatation represent the key features of LV remodeling after AMI and typically occurs in different subgroups of patients after AMI [6]. Initial studies showed that early LV dilatation with increased LV volume were strong predictors of heart failure and mortality [4–6]. Subsequent studies showed, that late LV dilatation was also associated with increased major cardiac events and cardiac death [7]. Bolognese et al. studied the prognostic impact of early and late LV dilatation after AMI and found no difference in mortality between the two dilatation types [6]. However, early LV dilatation was variably defined in the literature as increased LV end-diastolic volume at 1 to 6 months after AMI compared to the pre-discharge study [6, 8–10]. This variable definition of early dilatation may explain the absent prognostic importance of early and late LV dilatation in the past literature [6, 9]. Furthermore, this definition does not consider patients with early LV dilatation, which is already present on the pre-discharge study.

We hypothesized that early LV dilatation present at pre-discharge is associated with an adverse prognosis, since this remodeling type occurs immediately after infarction which may represent the more profound myocardial damage compared to the remodeling type with initial normal LV volume and delayed LV enlargement.

This longitudinal study analyzed the long-term prognostic impact of early pre-discharge and late LV dilatation up to > 15 years in patients with first ST-elevation myocardial infarction (STEMI) treated by PCI and contemporary medical therapy.

## Materials and methods

### Patients

**Fig. 1:**
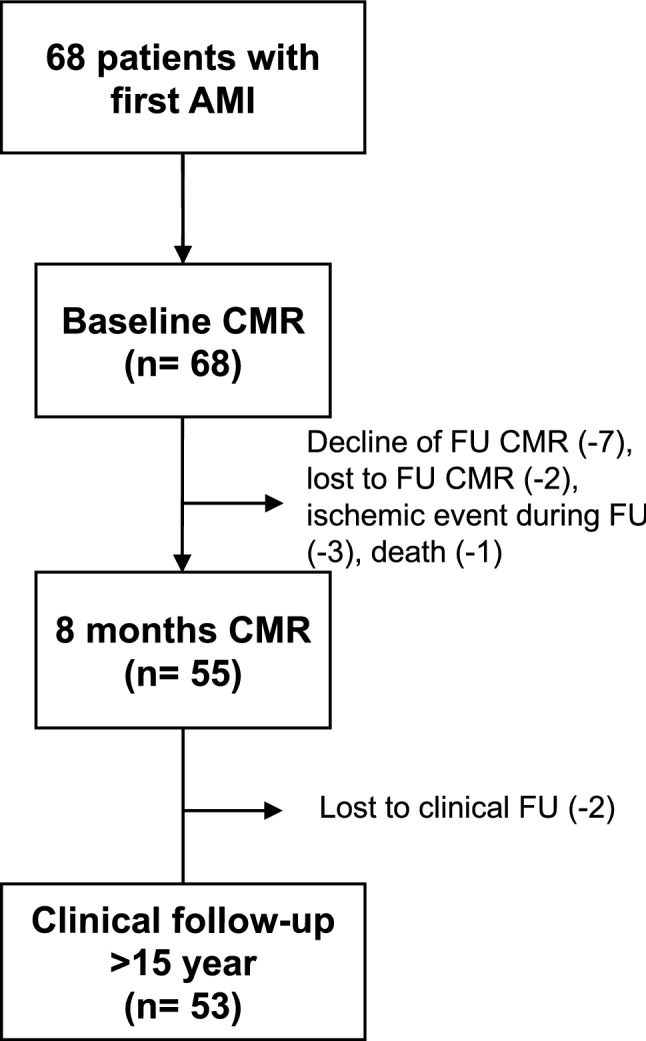
Patient flowchart. Sixty-eight consecutive patients with first acute myocardial infarction (AMI) were prospectively enrolled. Baseline cardiac magnetic resonance (CMR) was obtained in all patients, whereas follow-up (FU) CMR was available in 55 patients. Long-term clinical follow-up was available in the final study population of 53 patients

The ethics committee of the Hamburger Ärztekammer approved the study (reference number PV3451) and all patients gave written informed consent. Sixty-eight consecutive patients with first STEMI were prospectively enrolled between April 2002 and June 2003. STEMI was defined by prolonged chest pain, peak creatine kinase MB more than twice the normal upper limit of 5 U/L, and 1.0 mm or more ST segment elevation in two or more leads on the initial 12-lead electrocardiography. Peak creatine kinase (CK), creatine kinase myocardial band were obtained. Patients were treated by primary percutaneous coronary intervention (PCI) (n = 58) or facilitated PCI after thrombolysis (n = 10). Adequate restoration of blood flow of the infarct-related artery was determined on coronary angiograms by two experienced observers (K.M. and G.K.L., experience > 15 years to read coronary angiograms) in consensus reading using the Thrombolysis in Myocardial Infarction (TIMI) trial criteria. The total ischemic time was recorded defined as the time between beginning of chest pain and reperfusion of the infarct-related artery [11]. All patients obtained baseline pre-discharge CMR at 5 ± 3 days after AMI. Follow-up CMR was performed in 55 patients at 8 ± 3 months after AMI. The final study population included 53 of 55 patients (96%) with baseline and follow-up CMR and available long-term follow-up > 15 years. The reasons for missed CMR or unavailable clinical follow-up are given in Fig. 1.

### CMR protocol

CMR was performed in identical fashion at baseline and follow-up after AMI using a 1.5-T scanner (Vision; Siemens, Erlangen, Germany). The imaging protocol included standard steady-state free-precession cine CMR in short axis for LV volume and mass measurement using the following parameters: voxel size 1.37 × 1.37 × 8 mm^3^, gap 2 mm, 9–11 slices for full LV coverage, echo time = 1.8 ms, time to repetition = 3.6 ms. Inversion recovery LGE imaging was performed 10 min after injection of a dose of 0.1 mmol/kg gadopentetate dimeglumine (Magnevist; Schering, Berlin Germany) on end-diastolic short-axis using the following parameters: Voxel size 0.37 × 2.0 × 6 mm^3^, echo time = 3.4 ms, time to repetition = 7.6 ms. Inversion time 220–300 ms to null the signal intensity of normal myocardium. LGE images were obtained every other heartbeat to give time for more complete inversion recovery.

### CMR data analysis

Two investigators (E.T. and M.S., 8 and 5 years of experience in reading CMR, respectively) independently and blindly analyzed each CMR set in random order using certified software (cmr42, Circle Cardiovascular Imaging Inc., Calgary, Canada). CMR parameters were indexed to the calculated body surface area (BSA) and are given as the mean of the two observers. Measurements of LV volumes and LV mass was performed in standard fashion on short-axis cine images [12]. Infarct sizes were measured on three (basal, mid and apical) LV slices using a threshold method by placing a large region of interest into remote normal myocardium. The threshold was set to > 2 standard deviations (SD) above the mean values of these reference regions of interest for LGE images for all time points after AMI [13]. Mean infarct size was calculated as the total amount of enhanced myocardium above the threshold and is given in percent of LV area (%LV) by dividing infarct area by the total LV area on the three slices [14]. Microvascular obstruction was identified and measured on LGE images as a region of subendocardial hypoenhancement < 2 SD within the enhanced myocardium, which was previously determined as described above [8].

### Definition of early and late LV dilatation

**Fig. 2:**
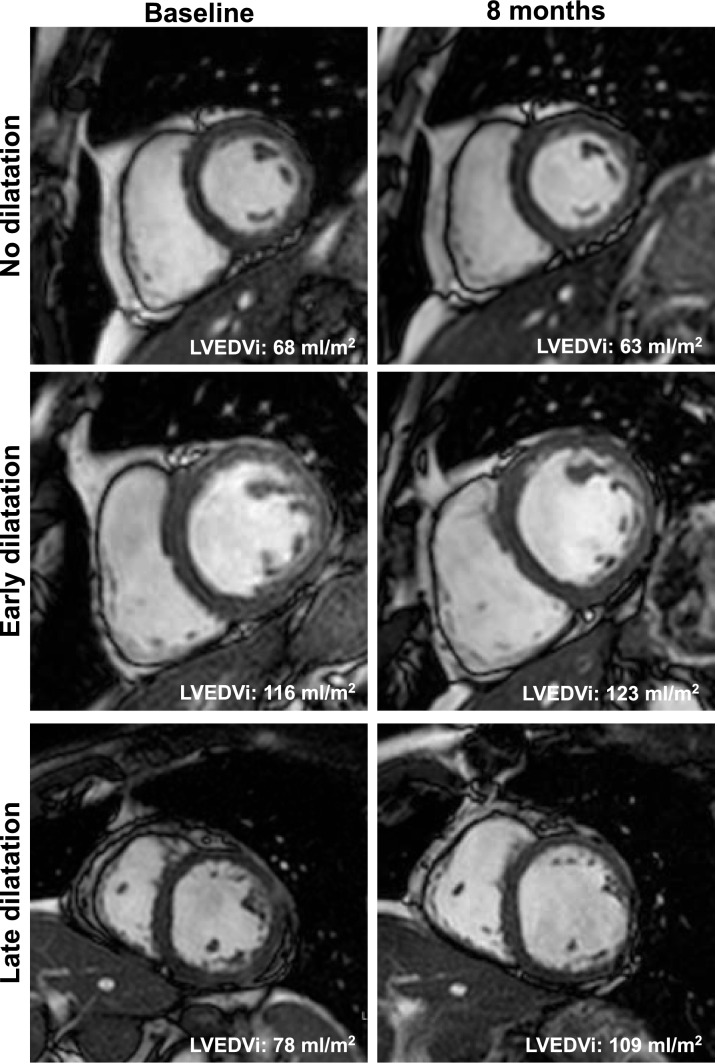
Diastolic cine CMR short axis images of patients with different LV dilatation types after AMI. The patient without dilatation had normal left ventricular end-diastolic volume index (LVEDVi) at baseline and 8 months follow-up. The patient with early dilatation had increased LVEDVi at baseline and 8 months follow-up, whereas the patient with late dilatation had normal LVEDVi at baseline, but increased LVEDVi at 8 months follow-up

Early LV dilatation was defined as increased left ventricular end-diastolic volume index (LVEDVi) at pre-discharge baseline CMR exceeding the upper 95% confidence interval of published normal LVEDVi with > 97 ml/m^2^ for males and > 90 ml/m^2^ for females, which were valid at the time of data acquisition [9]. Late dilatation was defined as an initially normal LVEDVi, which increased ≥ 20% within the first 8 months after AMI [6]. LVEDVi between the 5% and 95% CI of published normal values was interpreted as normal LVEDVi [9]. CMR images of patients with different types of dilatation are shown in Fig. 2.

### Outcome measures

Survival information was obtained by mail, telephone contact or from the public death registry. Information about major cardiac events (MACE) was obtained by mail or telephone contact. MACE was defined as resuscitation for sustained ventricular tachycardia and primary or secondary implantable cardioverter-defibrillator.

### Statistical analysis

Statistical analysis was performed using SPSS for Windows, version 23.0 (IBM SPSS Statistics, Armonk, NY). Categorical data are presented as absolute numbers and percentage and were compared using a Fisher’s exact or chi-squared test, as appropriate. Continuous data were presented as median and first [Q1] and third [Q3] quartiles and were compared using Mann–Whitney U test. Outcome was assessed using Kaplan–Meier graphs and a Cox proportional hazards model to compare groups and to obtain hazard ratios with Wald confidence intervals. A univariate analysis was performed to identify predictive parameters of survival and the combined endpoint of survival and MACE. In this univariate analysis, all parameters were selected that had a *P* < 0.05 on testing between dilatation type groups. We also performed a univariate analysis, that included all parameters which are expected to be relevant based of what is known in the literature. Then, a multivariate Cox model was performed with stepwise forward selection including all parameters, which had a *P*-value < 0.2 in the univariate testing to identify independent predictive parameter. All tests were two-sided and a *P*-value < 0.05 was considered significant.

## Results

### Baseline patient characteristics

No differences were present in respect to age, sex and body surface area between patients with no dilatation (n = 35, 66%), early (n = 7, 13%) and late dilatation (n = 11, 21%, Table 1). Patients with early LV dilatation had a higher rate of anterior infarcts (86%) compared to patients with no dilatation (37%, *P* < 0.05). No differences were observed between the groups in respect to the revascularization strategy by either primary PCI or facilitated PCI after thrombolysis (Table 1). Successful revascularization with a TIMI flow of 2 or greater was achieved in 94% of patients without significant differences in patients with no, early and late dilatation. Patients with early dilatation had largest LVEDVi and lowest ejection fraction of all three groups (Table 1). Median total ischemic time of all patients was 8.7 h (Q1-3, 3.6–24.2 h) and showed no significant difference between groups with different remodeling type. Patients with late dilatation had similar LVEDVi at baseline with 75 ml/m^2^ (Q1-Q3, 56–81 ml/m^2^) compared to patients with no dilatation with 76 ml/m^2^ (Q1-Q3, 66–82 ml/m^2^, *P* = 0.611). The LV mass index was higher in patients with early dilatation (95 g/m^2^, Q1-Q3, 77–138 g/m^2^, *P* < 0.05) and in patients with late dilatation (89 g/m^2^, Q1-Q3, 73–96 g/m^2^, *P* < 0.05) compared to patients with no dilatation (69 g/m^2^, Q1-Q3, 67–87 g/m^2^). The infarct sizes of patients with early and late dilatation were higher with 26%LV (Q1-Q3, 11–31%LV) and 26%LV (Q1-Q3, 17–31%LV, *P* < 0.01), respectively, compared to patients with no dilatation with 17%LV (Q1-Q3, 9–20%LV). No difference in infarct size was present between patients with early and late dilatation, *P* = 0.724). Patients with early dilatation had the largest MO size with 5.1%LV (Q1-Q3, 4.7–5.1%LV), however, MO was present in only 2 of 7 patients (29%) of this group.

**Table 1:** Clinical, infarct and CMR characteristics

Characteristic	No dilatation (n = 35, 66%)	Early dilatation (n = 7, 13%)	Late dilatation (n = 11, 21%)
Clinical			
Age, years	54 (46–66)	60 (37–65)	58 (47–67)
Male sex, n (%)	30 (86)	7 (100)	9 (82)
Body surface area, m^2^	1.9 (1.8–2.1)	1.9 (1.8–2.0)	2.0 (1.9–2.1)
Infarct characteristics			
Peak CK, U/L	704 (295–1179)	863 (604–1902)	1152 (424–1569)
Peak CK-MB, U/L	92 (38–135)	80 (72–99)	138 (53–180)
Anterior infarct, n (%)	13 (37)	6 (86) *	7 (64)
Angiography			
Infarct-related artery, n (%)			
LAD, n (%)	13 (37)	6 (86)	7 (64)
CFX, n (%)	7 (20)	1 (14)	2 (18)
RCA, n (%)	15 (43)	0 (0)	2 (18)
Primary PCI, n (%)	30 (86)	5 (71)	10 (91)
Facilitated PCI after thrombolysis, n (%)	5 (14)	2 (29)	1 (9)
No perfusion before PCI (TIMI ≤ 1), n (%)	22 (63)	5 (71)	9 (82)
Successful revascularization (TIMI ≥ 2), n (%)	33 (94)	6 (86)	11 (100)
Total ischemic time (h)	8.8 (3.3–23)	8.0 (3.1–40)	7.9 (4.3–25.5)
Secondary prevention medication			
ACEI or ARB, n (%)	18 (51)	6 (86)	9 (82)
Beta-Blocker, n (%)	32 (91)	6 (86)	8 (73)
Diuretics, n (%)	5 (14)	3 (43)	2 (18)
Statins, n (%)	13 (37)	6 (86)	11 (100)
Aspirin/Clopidogrel, n (%)	35 (100)	16 (100)	11 (100)
CMR parameters			
Ejection fraction, %	55 (51–62)	42 (29–60)	49 (35–57)^‡^
LVEDVi, ml/m^2^	76 (66–82)	106 (101–115)^†^	75 (56–81) ^¶^
LVESVi, ml/m^2^	32 (27–37)	67 (43–88)^†^	40 (37–52) ^||^
LV mass index, g/m^2^	69 (67–87)	95 (77–138)*	89 (73–96)^‡^
Infarct size, %LV	17 (9–20)	26 (11–31)	26 (17–31)^§^
MO size, %LV	0.7 (0.1–2.5)	5.1 (4.7–5.1)*	2.6 (2–3.4)
Presence of MO, n (%)	11 (31)	2 (29)	7 (64)

Values are presented as n (%) for categorical and median [first (Q1) and third (Q3) quartiles] for continuous data

^*^P < 0.05 or ^†^*P* < 0.01 for no dilatation vs. early dilatation

^‡^*P* < 0.05 or ^§^*P* < 0.01 for no dilatation vs. late dilatation

^||^*P* < 0.05 or ^¶^*P* < 0.01 for early vs. late dilatation

*ACEI* Angiotensin Converting Enzyme Inhibitor, *ARB* Angiotensin Receptor Blocker, *CFX* circumflex artery, *CK* creatine kinase, *CK-MB* creatine kinase myocardial band, *CMR* cardiac magnetic resonance, *LVEDVi* left ventricular end-diastolic volume index, *LVESVi* left ventricular end-systolic volume index, *LAD* left anterior descending artery, *LV* left ventricular, *MO* microvascular obstruction, *PCI* percutanous coronary intervention, *RCA* right coronary artery, *TIMI* Thrombolysis in Myocardial Infarction

### Differences between patients with early and late dilatation

There were no differences in respect to clinical, infarct or angiographic characteristics between patients with early and late dilatation (Table 1). Also, secondary prevention medication was not different between the two groups. According to the applied definition of early dilatation LVEDVi and LVESVi were higher in patients with early dilatation. No other CMR parameter was different between the two groups including ejection fraction, LV mass index, infarct size and size or presence of microvascular obstruction.

### Outcome

**Fig. 3:**
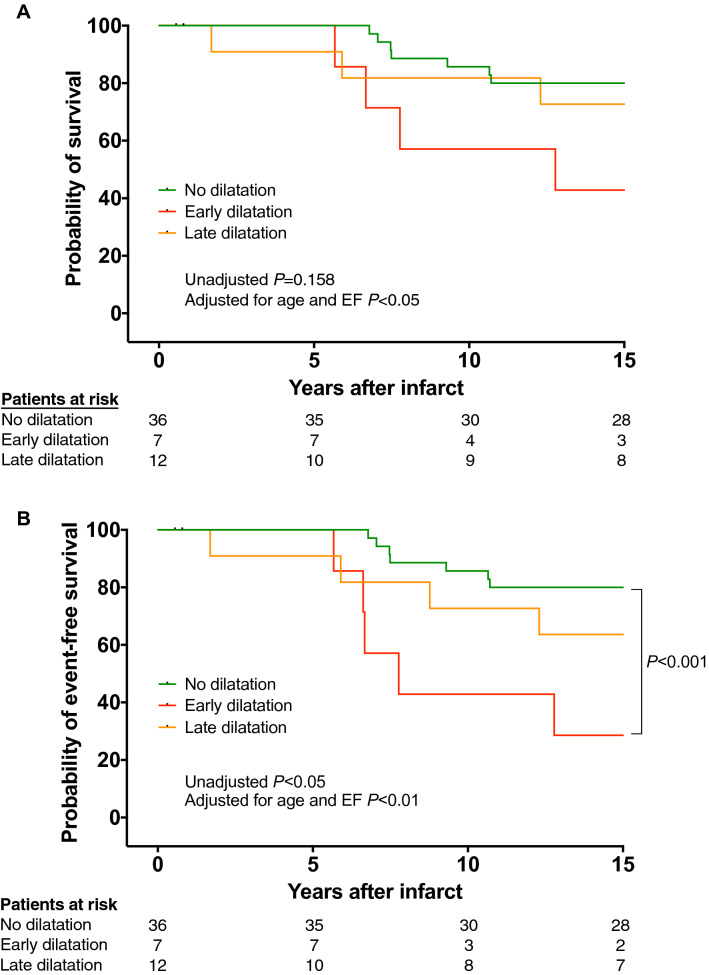
Clinical outcome stratified by LV dilatation type for all-cause mortality **a** and the composite end point including death and MACE **b** during long follow-up > 15 years. Patients with early dilatation had highest mortality rate with 57%, which was significant after adjustment for age and ejection fraction compared to patients with no dilatation with 26% (*P* < 0.05). Event-free survival was significant different between patients with early and no dilatation in the unadjusted and the adjusted model **b**. Note, that most events occurred after a follow-up > 5 years

Fourteen patients (26%) died during long-term follow-up and 16 (30%) experienced the composite end point of death and MACE including one patient with resuscitation for sustained ventricular tachycardia and one patient with primary implantable cardioverter-defibrillator. Patients with early dilatation had the highest mortality rate (57%) compared to patients with late dilatation (27%) and to patients with no dilatation (26%) (Fig. 3). Univariate Cox analysis based on parameters, which showed significant differences between dilatation type groups revealed that age (*P* < 0.001), baseline ejection fraction (*P* < 0.01), early dilatation (*P* = 0.056) and facilitated PCI after thrombolysis versus primary PCI (*P* = 0.172) were potential predictors of death and MACE (Table 2a + b). Univariate analysis that included all parameters, which are expected to be relevant based of what is known in the literature revealed that also LVESVi was a potential predictor of death and MACE (*P* < 0.05, Table 2a + b). All potential parameters were included in the multivariate Cox model. Facilitated versus primary PCI (*P* = 0.507) and LVESVi (*P* = 0.564) dropped out in the multivariate model whereas age (*P* < 0.001), ejection fraction at baseline (*P* < 0.01) and early dilatation (*P* < 0.05) were independent predictors of death. After adjustment for age and ejection fraction early dilatation was a significant predictor of mortality (*P* < 0.05, Fig. 3a) with a hazard ratio of 2.2 (95% confidence interval: 1.2 to 7.9). Early dilatation was a potent predictor for the combined endpoint death and MACE in the unadjusted (*P* < 0.05) and in the age and ejection fraction adjusted model (*P* < 0.01, Fig. 3b).

**Table 2:** Results of Cox-Regression: dead–local p-values

A							
		Univariate Cox-Regression			Final multivariate Cox-Regression		
Parameter	Level	β Coefficient	SE	*P*	β Coefficient	SE	*P*
No dilatation	Early dilatation	206.054	0.66010	0.0566	0.80306	0.74281	0.006
No dilatation	Late dilatation	−126.336	100.294	0.7151	−169.526	111.746	0.036
Age, years		0.09635	0.02610	< 0.001	0.12444	0.03524	< 0.001
Peak CK, U/L		0.000136	0.000281	0.6279	Excluded*	–	–
Anterior infarct	Posterior infarct	0.72542	0.54803	0.2156	Excluded*	–	–
Facilitated PCI after thrombolysis	Primary PCI	0.86115	0.58477	0.172	Excluded*	–	–
No perfusion before PCI (TIMI ≤ 1)	Perfusion before PCI (TIMI > 1)	0.28515	0.58465	0.6257	Excluded*	–	–
Successful revascularization (TIMI ≥ 2)	Unsuccessful revascularization (TIMI < 2)	−1.510.641	1882	0.9936	Excluded*	–	–
Total ischemic time (h)		−0.0003490	0.00506	0.9450	Excluded*	–	–
Ejection fraction, %		−0.05336	0.01991	0.003	−0.08677	0.03228	0.002
LVESVi, ml/m^2^		0.04127	0.01956	0.0349	Excluded*	–	–
LV mass index, g/m^2^		−0.0002851	0.00894	0.9746	Excluded*	–	–
Infarct size, %LV		0.01386	0.02821	0.6232	Excluded*	–	–
MO size, %LV		0.27337	0. 22,782	0.2302	Excluded*	–	–
Presence of MO	No MO	−0.46786	0.51800	0.3664	Excluded*	–	–

B							
		Univariate Cox-Regression			Final multivariate Cox-Regression		
Parameter	Level	β Coefficient	SE	*P*	β Coefficient	SE	*P*
No dilatation	Early dilatation	156.843	0.57312	0.0062	206.054	0.66010	0.0018
No dilatation	Late dilatation	0.59540	0.61256	0.3311	−126.336	100.294	0.2078
Age, years		0.08812	0.02344	0.0002	0.12157	0.03243	0.0002
Peak CK, U/L		−0.0000667	0.000915	0.9418	Excluded*	–	–
Anterior infarct	Posterior infarct	−1.790.311	6478	0.9978	Excluded*	–	–
Facilitated PCI after thrombolysis	Primary PCI	106.306	0.53587	0.0473	Excluded*	–	–
No perfusion before PCI (TIMI ≤ 1)	Perfusion before PCI (TIMI > 1)	−0.52072	141.947	0.7137	Excluded*	–	–
Successful revascularization (TIMI ≥ 2)	Unsuccessful revascularization (TIMI < 2)	−1.619.576	6469	0.9980	Excluded*	–	–
Total ischemic time (h)		0.01221	0.01165	0.2309	Excluded*	–	–
Ejection fraction, %		−0.05404	0.01844	0.0034	−0.08271	0.02876	0.0040
LVESVi, ml/m^2^		0.16621	0.09344	0.0753	Excluded*	–	–
LV mass index, g/m^2^		−0.02215	0.05100	0.6641	Excluded*	–	–
Infarct size, %LV		0.07029	0.07155	0.3259	Excluded*	–	–
MO size, %LV		0.26779	0.52342	0.6089	Excluded*	–	–
Presence of MO	No MO	−0.57633	141.497	0.6838	Excluded*	–	–

*SE* Standard Error

^*^Excluded in the backward selection process of the multivariate Cox-Regression (P > 0.2) as in Table 1

## Discussion

This longitudinal CMR study focused on the long-term prognostic impact of early pre-discharge and late LV dilatation in patients with first STEMI treated by PCI during > 15 years follow-up. The major findings of our study were: First, patients with early pre-discharge dilatation had highest mortality (57%) during long-term follow-up, whereas patients with late or with no dilatation had similar mortality, illustrating the high risk of early dilatation and the more benign nature of late dilatation. Second, early dilatation was a potent independent predictor of death and MACE, pointing out the potential to timely identify patients with poor prognosis early after STEMI based on LV dilatation. Third, enzyme release and infarct size by CMR were not different in patients with early and late LV dilatation, suggesting that the amount of acute myocardial injury has no direct impact on the LV dilatation type.

### Prognostic implications of early and late LV dilatation

Our study showed that early pre-discharge LV dilatation was associated with high mortality rate (57%) during follow-up compared to 26% in patients with no LV dilatation. Furthermore, early dilatation was a potent predictor of death after adjustment for ejection fraction and age, which were also associated with death in the univariate Cox model. Other parameters, which were associated with reduced prognosis in previous studies, such as infarct size [10], presence of microvascular obstruction [15] or increased LV mass index [11] were not predictive of death in this longitudinal study. Interestingly, patients with late LV dilatation had similar mortality with 27% compared to patients with no LV dilatation (20%), indicating that late LV dilatation is a post-infarction complication with lower prognostic relevance in post-infarction patients who receive a contemporary medical anti-remodeling therapy.

### Previous studies about prognosis of dilatation type

Bolognese et al. analyzed the impact of the LV dilatation type on prognosis after AMI treated by primary PCI [7]. This study showed reduced prognosis of patients with LV remodeling compared to patients without remodeling, however, no survival differences were found between patients with early and late LV dilatation. The main discrepancies between our and the aforementioned study were the long follow-up > 15 year and the higher mortality rate of 26% in the current study compared to a follow-up of 5.1 ± 1.2 years and a mortality rate of 13% in Bolognese et al. study [7]. Our Kaplan–Meier analysis revealed, that most deaths occurred after a follow-up > 5 years after first AMI, indicating that a long follow-up is necessary to study the prognostic implications in the era of PCI and contemporary medical therapy. Additionally, Bolognese et al. used a different definition of early LV dilatation compared to our study, which may explain the contradictory findings. Bolognese et al. defined early dilatation as an increased end-diastolic volume ≥ 20% at 1-month compared to baseline at 24 h after AMI [7]. Conversely, we defined early LV dilatation as an increased LVEDVi at 5 days after AMI exceeding the upper 95% confidence interval of published normal values. Beside the strong prognostic impact our definition has the advantage that early dilatation can be promptly detected after infarction to initiate a timely and more intensive medical treatment, whereas Bolognese et al. definition requires an additional follow-up at 1 month after AMI.

### Factors that promote early or late LV dilatation

The current data did not reveal difference in clinical and CMR parameters between patients with early and late dilatation. Especially, the infarct characteristics, such as enzyme release, infarct size and the revascularization strategy and success were not different between the two groups. Therefore, other factors must be responsible for the differential occurrence of early and late dilatation. It is possible, that patients with early or late dilatation had a longer the time to revascularization compared to patients with no remodeling. However, the total ischemic time was not different between groups.

### Limitations

One limitation of our study is the relatively small number of patients with long follow-up, which may have affected the identification of other predictive factors associated with poor prognosis after AMI. Furthermore, we had to apply a stringent cut-off for the definition of late dilatation by using the upper 95% CI, since our unique long-time follow-up population is inherently relatively small. Nevertheless, milder degrees of early dilatation (e.g. the upper quartile) could also be associated with poor outcome in a larger study population.

Another limitation is, that comprehensive information on CV risk factors and comorbidity and their changes during the follow-up period were not available.

## Conclusions

Patients with early pre-discharge LV dilatation, defined as increased LVEDVi at 5 days after infarction had highest mortality of 57% during long-term follow-up. Furthermore, early dilatation was an independent predictor of death and MACE, underlining the importance to reduce this post-infarction complication. Conversely, late LV dilatation had similar outcome compared to patients with no dilatation, indicating that this dilatation type is less prognostic relevant in the era of infarct treatment by PCI and contemporary medical therapy.

### Translational outlook

The current study revealed that infarct characteristics, such as infarct size by CMR, enzyme release and the revascularization strategy and success were not different between patients with early and late LV dilatation. Therefore, other factors must be responsible for early LV dilatation, such as time to revascularization or amount of collateral flow during infarction. Further studies are needed to confirm occurrence and prognostic relevance of early pre-discharge LV dilatation after AMI in patients treated by the current standard of care and to better understand the causes of this LV dilatation type to prevent this deleterious complication in future.

## Supplementary Information

Below is the link to the electronic supplementary material.

Supplementary file1 (sav 11 KB)

## Data Availability

All data are available in a supplementary file for statistical review.
